# Medical accuracy of artificial intelligence chatbots in oncology: a scoping review

**DOI:** 10.1093/oncolo/oyaf038

**Published:** 2025-04-26

**Authors:** David Chen, Kate Avison, Saif Alnassar, Ryan S Huang, Srinivas Raman

**Affiliations:** Princess Margaret Hospital Cancer Centre, Radiation Medicine Program, Toronto, ON M5G 2C4, Canada; Temerty Faculty of Medicine, University of Toronto, Toronto, ON M5S 3K3, Canada; Princess Margaret Hospital Cancer Centre, Radiation Medicine Program, Toronto, ON M5G 2C4, Canada; Department of Systems Design Engineering, University of Waterloo, Waterloo, ON N2L 3G1, Canada; Princess Margaret Hospital Cancer Centre, Radiation Medicine Program, Toronto, ON M5G 2C4, Canada; Department of Systems Design Engineering, University of Waterloo, Waterloo, ON N2L 3G1, Canada; Princess Margaret Hospital Cancer Centre, Radiation Medicine Program, Toronto, ON M5G 2C4, Canada; Temerty Faculty of Medicine, University of Toronto, Toronto, ON M5S 3K3, Canada; Princess Margaret Hospital Cancer Centre, Radiation Medicine Program, Toronto, ON M5G 2C4, Canada; Temerty Faculty of Medicine, University of Toronto, Toronto, ON M5S 3K3, Canada; Department of Radiation Oncology, University of Toronto, Toronto, ON M5T 1P5, Canada; Department of Radiation Oncology, BC Cancer, Vancouver, BC V5Z 1G1, Canada; Division of Radiation Oncology, University of British Columbia, Vancouver, BC V5Z 1M9, Canada

**Keywords:** artificial intelligence, chatbot, clinical decision support, medical accuracy

## Abstract

**Background:**

Recent advances in large language models (LLM) have enabled human-like qualities of natural language competency. Applied to oncology, LLMs have been proposed to serve as an information resource and interpret vast amounts of data as a clinical decision-support tool to improve clinical outcomes.

**Objective:**

This review aims to describe the current status of medical accuracy of oncology-related LLM applications and research trends for further areas of investigation.

**Methods:**

A scoping literature search was conducted on Ovid Medline for peer-reviewed studies published since 2000. We included primary research studies that evaluated the medical accuracy of a large language model applied in oncology settings. Study characteristics and primary outcomes of included studies were extracted to describe the landscape of oncology-related LLMs.

**Results:**

Sixty studies were included based on the inclusion and exclusion criteria. The majority of studies evaluated LLMs in oncology as a health information resource in question-answer style examinations (48%), followed by diagnosis (20%) and management (17%). The number of studies that evaluated the utility of fine-tuning and prompt-engineering LLMs increased over time from 2022 to 2024. Studies reported the advantages of LLMs as an accurate information resource, reduction of clinician workload, and improved accessibility and readability of clinical information, while noting disadvantages such as poor reliability, hallucinations, and need for clinician oversight.

**Discussion:**

There exists significant interest in the application of LLMs in clinical oncology, with a particular focus as a medical information resource and clinical decision support tool. However, further research is needed to validate these tools in external hold-out datasets for generalizability and to improve medical accuracy across diverse clinical scenarios, underscoring the need for clinician supervision of these tools.

**Implications for practice**

Artificial intelligence chatbots are promising but untested tools in oncology that may enhance clinical decision support through applications in symptom screening, diagnosis, management, and as health information resources across multiple cancer types and care contexts. Emerging strategies like prompt engineering and fine-tuning show potential to improve chatbot medical accuracy. However, further evaluation is required to address concerns about output accuracy, reliability, and scope limitations, emphasizing the critical need for clinician oversight to ensure safe and effective integration into oncology workflows.

## Introduction

The evolution of artificial intelligence (AI) and natural language processing (NLP) technologies has motivated the design of algorithms capable of generating text with human-like competency.^1-3^ Within the field of artificial intelligence, machine learning uses statistical models to learn patterns in data and predict outcomes based on the training data.^4^ To extend the ability of machine learning to extract knowledge from data, modern AI architectures have focused on the design of deep learning models that require less human intervention to learn high-dimensional patterns within data.^5^ Natural language processing, another branch of AI, focuses on bridging the gap between natural languages and computers to allow computers to understand, interpret, and generate human-like text.^6^

Chatbots, also known as conversational large language models (LLM), serve as the intersection of deep learning and natural language processing in the field of artificial intelligence. Chatbots mimic human natural language understanding to intake text and output context-relevant text based on the input.^7,8^ The specific computational algorithm differs based on the chatbot’s application but can commonly include the Sequence to Sequence model, the Pattern Matching Algorithm, and the Naive Bayes Algorithm. With the advent of transformer architecture, which uses encoder-decoder mechanisms, chatbots have significantly improved in handling complex language tasks and generating more natural responses.^9,10^ LLMs, such as the Generative Pre-trained Transformer (GPT-3), are advanced AI systems trained on vast amounts of text data using deep learning-based transformer architectures and can generate human-like responses, answer questions, and perform various specialized language-related tasks.^11,12^

Although limitations to early natural language processing models, such as biased training data, ethical concerns, and inaccurate outputs, prevented their widespread adoption in medicine, the advent of deep learning-based conversational agents capable of self-learning enables near-human levels of language understanding.^13^ In this context, self-learning refers to the model’s ability to improve and adapt without explicit human intervention.^14^ These advancements marked a significant step forward for AI in medicine, facilitating the potential to improve diagnostic accuracy, perform risk assessments, and serve as a tool for patient education and information retrieval.^15-17^ Furthermore, chatbots’ ability to manage complex dialogue and generate contextually relevant responses in multiple languages serves as another useful competency in clinical practice.^18^ Moreover, when applied as a health information resource, chatbots may provide 24/7 access to cancer-related services and can help individuals with low computer and health literacy to readily locate and understand cancer-related information, regardless of their proximity to medical services.^19^

Throughout a patient’s medical journey, chatbots can provide support in a variety of ways, including symptom screening, diagnosis assistance, remote monitoring, and post-treatment care. In complex cancer care settings, chatbots can uniquely aid in symptom screening and diagnosis as a clinical decision support tool,^20,21^ foster remote patient monitoring by providing access to care instructions and educational information,^22,23^ and be integrated as a health promotion tool for cancer survivorship.^24^ These features have the potential to reduce operating costs, increase workflow efficiencies, and save time for healthcare professionals by automating routine, laborious tasks related to cancer care.^25^

The medical accuracy of the LLM-based chatbots is a fundamental measure to assess the efficacy of this promising technology before its integration into clinical workflows. This scoping review aims to survey the landscape of LLM-based chatbot in oncology and their performance across diverse NLP tasks. Our review also aims to identify trends, challenges, and opportunities of large language model research in oncology.

## Methods

**Figure 1.**
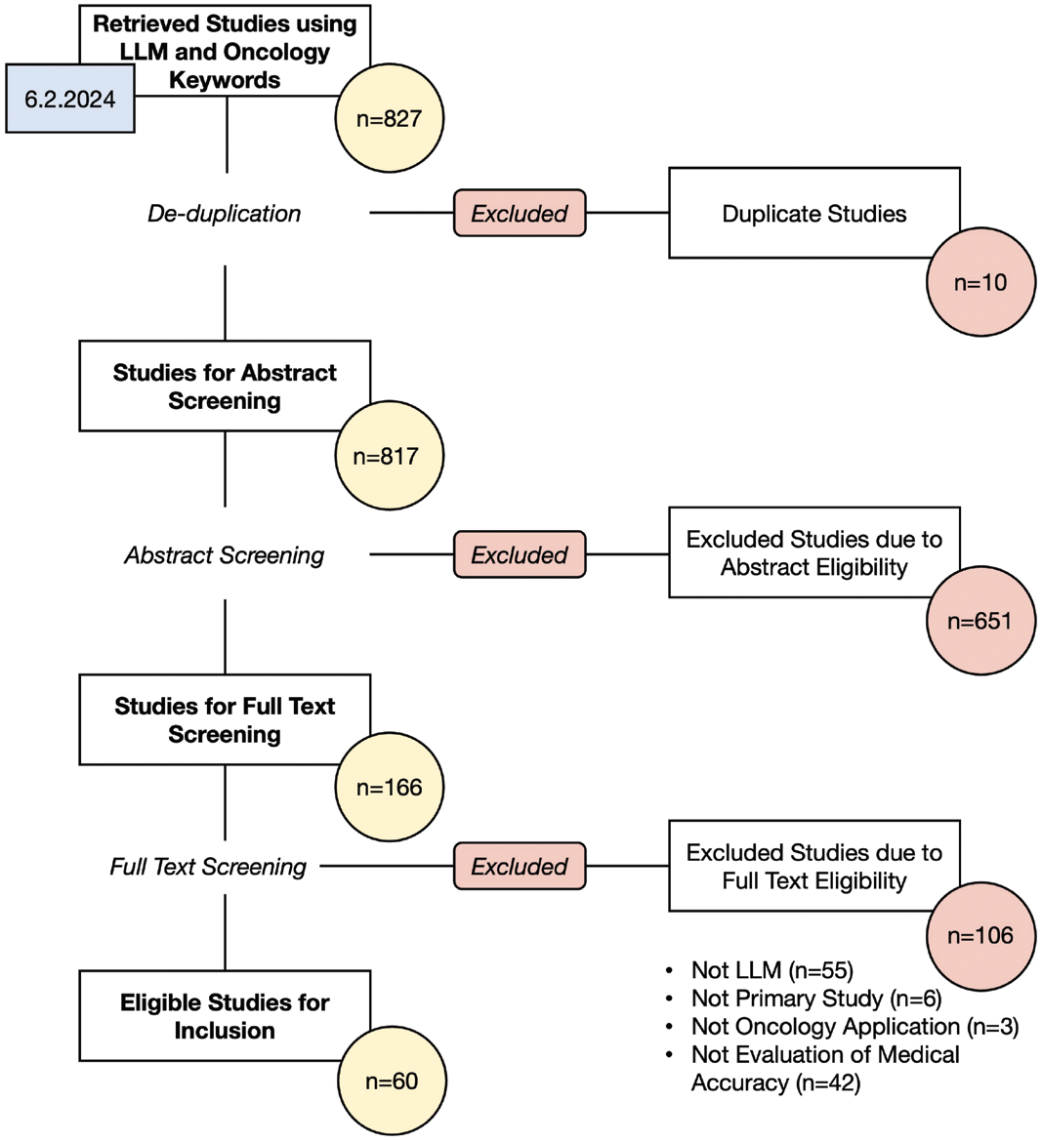
Search and filtering strategy used to select LLM-based chatbot studies evaluating medical accuracy for inclusion in this review.

We conducted a scoping literature search on OVID Medline on June 2, 2024 using MeSH and non-MeSH keywords related to oncology (“neoplasms,” “cancer,” “onco,” “tumor”) and generative large language models (“natural language processing,” “artificial intelligence,” “generative,” “large language model”). Articles that were not published in English language, published before 2000, or not published in peer-reviewed journals were excluded. Search was limited to articles published in the year 2000 or later given that large language model-based chatbotsm, such as BERT and GPT, were largely an advancement that emerged in the late 2010s. This search criteria yielded 827 articles for screening. Duplicate articles were removed (*n* = 10), leading to 817 articles for manual screening. Through dual reviewer screening, we conducted abstract screening followed by full-text screening, including articles that evaluated an LLM, were primary research studies, applied in oncology settings, and evaluated medical accuracy as a health information resource or clinical decision support tool. Articles that evaluated a natural language processing-based algorithm that did not involve a deep learning model trained on large text corpora, were secondary research articles, were applied in only non-oncology settings, and did not evaluate and report medical accuracy were excluded. This search yielded 60 studies that met the study inclusion and exclusion criteria. Supplementary Table 2 details the full OVID Medline search strategy. Figure 1 describes the PRISMA Scoping Review methodology for article screening and inclusion procedure based on the OVID Medline literature search. Supplementary Table 3 details the study’s adherence to the PRISMA Scoping Review reporting standard.

To examine the study characteristics and primary outcomes of the included studies, we extracted key study attributes from the article's full texts, including clinical domain, clinical application, large language model characteristics, study-reported outcomes, and study methodology. Study attributes with no available data were coded as “Not reported.” Study screening and data extraction were conducted in duplicate (K.A. and S.A.) and conflicts were resolved by consensus with discussion by a third reviewer (D.C.).

We conducted a narrative synthesis of the included studies by 3 team members (K.A., S.A., and D.C.). First, the synthesis of extracted data involved categorizing studies based on the specific model evaluated, the clinical domain, and common themes of benefits and risks based on outcomes reported by the studies. Second, we compared and contrasted the findings across studies, identifying recurring themes, patterns, and discrepancies. This involved examining the relationships between study characteristics (eg, population, setting) and reported outcomes. We also explored potential explanations for any observed variations in findings. Discussion of themes of strengths and limitations reported by the studies was conducted to appraise the included articles in the review. The synthesis process was iterative and involved ongoing discussion and refinement among the review team. This ensured a rigorous and transparent approach to synthesizing the diverse evidence and providing a comprehensive overview of the landscape of included studies.

## Results

Our study included 60 studies that met the inclusion and exclusion criteria, with 31 studies that evaluated LLM applications for clinical tasks in oncology (eg, diagnosis, management, screening) shown in Table 1 and 29 studies that evaluated LLMs on oncology-related examinations as a health information resource shown in Table 2.^7,26–37,39–85^ Additional information about study-specific methodology (dataset source, data type, and evaluation methodology) can be found in Supplementary Table 1. The included studies primarily evaluated the medical accuracy of ChatGPT-based applications in clinical oncology.

**Table 1.** Characteristics and primary outcomes of studies that evaluated LLM-based chatbot medical accuracy as a clinical decision support tool.

Study ID	Clinical domain	Clinical application	Baseline model	Baseline or fine-tuned LLM	Zero-shot or prompt engineered	LLM main outcomes
Shiraishi 2023^26^	Skin	Diagnosis	ChatGPT-4.0; Bard; Bing	Baseline	Zero-shot	Bing had an average accuracy of 58%. ChatGPT-4 and Bard refused to answer the clinical queries with images.
Cirone 2024^27^	Skin	Diagnosis	ChatGPT-4.0V; LLaVA	Baseline	Zero-shot	GPT-4V outperformed LLaVA in all examined areas; GPT-4V provided thorough descriptions of relevant ABCDE features
Yang 2024^28^	Orthopedic	Diagnosis	ChatGPT-3.5	Baseline	Prompt Engineered	Before few-shot learning, ChatGPT’s accuracy, sensitivity, and specificity were 0.73, 0.95, and 0.58, respectively; After, these metrics approached physician-level performance
Cozzi 2024^29^	Breast	Diagnosis	GPT-3.5; GPT-4.0; Bard	Baseline	Zero-shot	Agreement between human readers was almost perfect; Agreement between human readers and LLMs was moderate
Shifai 2024^30^	Skin	Diagnosis	ChatGPT-4.0V	Baseline	Zero-shot	ChatGPT Vision had a sensitivity of 32%, specificity of 40%, and overall diagnostic accuracy of 36%
Sievert 2024^31^	Head and Neck	Diagnosis	ChatGPT-4.0V	Baseline	Prompt Engineered	ChatGPT-4.0V scored lower than experts, acheiveing accuracy of 71.2% with an almost perfect intra-rater agreement (κ = 0.837)
Sievert 2024^31^	Thyroid	Diagnosis	ChatGPT-4.0	Baseline	Zero-shot	ChatGPT achieved a sensitivity of 86.7%, specificity of 10.7%, and accuracy of 68% when distinguishing between low-risk and high-risk categories
Wu 2024^32^	Thyroid	Diagnosis	ChatGPT-3.5; ChatGPT-4.0; Google Bard	Baseline	Zero-shot	ChatGPT 4.0 and Bard displayed higher intra-LLM agreement compared to ChatGPT-3.5; ChatGPT 4.0 had higher accuracy, sensitivity, and AUC than Bard
Ma 2024^33^	Esophagus	Diagnosis	ChatGPT-3.5	Baseline	Prompt Engineered	Accuracies were 0.925 to 1.0; Precisions were 0.934 to 0.969; Recalls were 0.925 to 1.0; F1-scores were 0.928 to 0.957
Rundle 2024^34^	Skin	Diagnosis	ChatGPT-4.0	Baseline	Zero-shot	Diagnosis accuracy was 66.7%; For malignant neoplasms, diagnosis accuracy was 58.8%; For benign neoplasms, diagnosis accuracy was 69.6%
Horiuchi 2024^35^	Central Nervous System	Diagnosis	ChatGPT-4.0	Baseline	Prompt Engineered	ChatGPT’s diagnostic accuracy was 50% for the final diagnosis; There were no significant differences in accuracy rates among anatomical locations
Tariq 2022^36^	Liver	Diagnosis	BERT	Baseline	Zero-shot	Malignant: 34%; Benign classification: 98%
Wang 2024^37^	Thyroid	Diagnosis	ChatGPT-4.0V	Baseline	Prompt Engineered	GPT-4 demonstrated proficiency in report structuring, professional terminology, and clarity of expression; GPT-4 showed limitations in diagnostic accuracy
Patel 2024^38^	Gynecologic	Genetic Counseling	ChatGPT-3.5	Baseline	Zero-shot	82.% “correct and comprehensive,” 15% “correct but not comprehensive,” 2.5% “partially incorrect” responses
Erdat 2024^39^	Pan-cancer	Management	ChatGPT-3.5; Bing	Baseline	Zero-shot	ChatGPT performed 5 of 9 correct protocols; Bing performed 4 of 9 correct protocols
Moll 2024^40^	Breast	Management	ChatGPT-3.5	Baseline	Zero-shot	ChatGPT’s answers were mostly correct but some contained inaccuracies; Patients expressed a preference for the presence of a physician
Gamble 2024^41^	Lung	Management	ChatGPT-3.5; ChatGPT-4.0	Baseline (GPT-3.5 and GPT-4.0); Fine-tuned (GPT-3.5)	Zero-shot	ChatGPT-3.5’s accuracy was 0.058 without guidelines and 0.42 with guidelines; ChatGPT-4 accuracy was 0.15 without guidelines and 0.66 with guidelines
Guo 2024^42^	Central Nervous System	Management	neuroGPT-X	Baseline; Fine-tuned	Zero-shot; Prompt Engineered	LLMs rated similarly to experts for accuracy, coherence, relevance, thoroughness, and performance; LLMs responsed faster than experts
Marchi 2024^43^	Head and Neck	Management	ChatGPT-3.5	Baseline	Zero-shot	ChatGPT performed better for primary treatment than for adjuvant treatment or follow-up recommendations
Choo 2024^44^	Colorectal	Management	ChatGPT-3.5	Baseline	Zero-shot	ChatGPT adhered to oncological principles in all cases; The concordance rate between chatGPT and the MDT board was 86.7%
Kuk 2024^45^	Geriatric Oncology	Management	ChatGPT-4.0	Baseline	Zero-shot	ChatGPT-4 identified medication-related side effects and suggested appropriate medications; GPT-4 was unable to suggest initial dosages of medications
Sorin 2023^46^	Breast	Management	ChatGPT-3.5	Baseline	Zero-shot	70% of ChatGPTs recommendations were comparable to recommendations by the tumor board
Schulte 2023^47^	Pan-cancer	Management	ChatGPT-3.5	Baseline	Zero-shot	Overall VTQ: 0.77
Griewing 2023^48^	Breast	Management	ChatGPT-3.5	Baseline	Prompt Engineered	Concordance: 58.8%
Chiarelli 2024^49^	Genitourinary	Screening	ChatGPT-3.5; ChatGPT-4	Baseline	Prompt Engineered	ChatGPT-4 performed better than ChatGPT-3.5 for accuracy, clarity, and conciseness; ChatGPT-4 exhibited high readability
Pereyra 2024^50^	Colorectal	Screening	ChatGPT-3.5	Baseline	Zero-shot	ChatGPT performed significantly lower than the physician groups, averaging a mean of 4.5/10 correct answers
Nguyen 2023^51^	Pan-cancer	Screening	ChatGPT-4.0; Bard	Baseline	Zero-shot; Prompt Engineered	ChatGPT and Bard performed similarly on OE prompts; ChatGPT performed better in SATA scenarios; Prompt engineering improved LLM outputs in OE prompts
Atarere 2024^52^	Colorectal	Screening	ChatGPT-4.0; BingChat; YouChat	Baseline	Zero-shot	ChatGPT and YouChat provided RA responses more often than BingChat for both question sets
Rao 2023^53^	Breast	Screening	ChatGPT-3.5; ChatGPT-4.0	Baseline	Zero-shot	Breast cancer screening: Free-text score: 1.83/2, Multiple-choice score: 88.9%; Breast pain: Free-text score: 1.125/2, Multiple-choice score: 58.3%
Ahmad 2023^54^	Pan-cancer	Management	BioBERT; RoBERTa	Baseline	Zero-shot	The proposed system achieved high accuracy and F1-score in predicting cancer treatment from EHRs
Benary 2023^55^	Pan-cancer	Management	ChatGPT-3.5; Galactica; Perplexity; BioMedLM	Baseline	Prompt Engineered	LLMs F1 scores of 0.04 (Biomed LM), 0.14 (Perplexity), 0.17 (ChatGPT), and 0.19 (Galactica); LLM-generated combined treatment options and clinical treatment options yielded median (IQR) scores of 7.5 (5.3-9.0) and 8.0 (7.5-9.5) points, respectively; Manually generated options reached a median score of 2.0 (1.0-3.0) points

**Table 2.** Characteristics and primary outcomes of studies that evaluated the medical accuracy of LLM-based chatbots as a health information resource.

Study ID	Clinical Domain	Clinical Application	Baseline Model	Baseline or Fine-tuned LLM	Zero-shot or Prompt Engineered	LLM Main Outcomes
Ostrowska 2024^56^	Head and Neck	Health Information	ChatGPT-3.5; ChatGPT-4; Google Bard	Baseline	Zero-shot	ChatGPT 3.5 scored highest in safety and Global Quality Score; ChatGPT 4.0 and Bard had lower mean safety scores
Szczesniewski 2024^57^	Genitourinary	Health Information	ChatGPT; Google Bard; Copilot	Baseline	Zero-shot	Each chatbot explained the pathologies, detailed risk factors, and described treatments well;Quality and appropriateness of the information varied
Iannantuono 2024^7^	Pan-cancer	Health Information	ChatGPT-3.5; ChatGPT-4.0; Google Bard	Baseline	Zero-shot	ChatGPT-4 and ChatGPT-3.5 had higher rates of reproducible, accurate, relevant, and readable responses compared to Google Bard
Lee 2023^58^	Head and Neck	Health Information	ChatGPT-4.0	Baseline	Zero-shot	ChatGPT-generated pre-surgical information performed similarly to publicly available websites; ChatGPT was preferred 48% of the time by H&N surgeons
Coskun 2023^59^	Genitourinary	Health Information	ChatGPT-3.5	Baseline	Zero-shot	ChatGPT responded to all queries; Calculated metrics indicated a need for improvement in its performance
Lum 2024^60^	Orthopedic	Health Information	ChatGPT-3.5; Bard	Baseline	Zero-shot	BARD answered more questions correctly than ChatGPT; ChatGPT performed better in sports medicine and basic science; BARD performed better in basic science
Dennstadt 2024^61^	Pan-cancer	Health Information	ChatGPT-3.5	Baseline	Zero-shot	94.3% of MC answers were considered “valid”; 48% of open-ended answers were considered “acceptable,” “good,” or “very good”
O’Hagan 2023^62^	Skin	Health Information	ChatGPT-4.0	Sexu	Zero-shot	Models were 100% appropriate for a patient-facing portal; EHR responses were appropriate 85%-100% of the time, depending on question type
Kuscu 2023^63^	Head and Neck	Health Information	ChatGPT-4.0	Baseline	Zero-shot	84.6% “comprehensive/correct,” 11% “incomplete/partially correct,” and 2.6% “incomplete/partially correct” responses
Deng 2024^64^	Breast	Health Information	ChatGPT-3.5; ChatGPT-4.0; Claude2	Baseline	Zero-shot	ChatGPT-4.0 outperformed ChatGPT-3.5 and Claude2 in average quality, relevance, and applicability; ChatGPT-4.0 scored higher than Claude2 in support and decision-making
Huang 2024^65^	Central Nervous System	Health Information	ChatGPT-4.0	Baseline	Zero-shot	ChatGPT-4.0 responses were consistent with guidelines; Responses ocasionally missed “red flag” symptoms; 50% of citations were deemed valid
Li 2024^66^	Cardio-oncology	Health Information	ChatGPT-3.5; ChatGPT-4.0; Google Bard; Meta Llama 2; Anthropic Claude 2	Baseline	Zero-shot	ChatGPT-4.0 performed best in producing appropriate responses; All 5 LLMs underperformed in the treatment and prevention domain
Hanai 2024^67^	Genitourinary	Health Information	ChatGPT-3.5	Baseline	Prompt Engineered	Both bots recommended non-pharmacological interventions significantly more than pharmacological interventions
Valentini 2024^68^	Orthopedic	Health Information	ChatGPT-3.5	Baseline	Zero-shot	The median score for ChatGPT’s answers was 18.3; 6 answers were very good, 9 were good, 5 were poor, and 5 were very poor
Yalamanchili 2024^69^	Pan-cancer	Health Information	ChatGPT-3.5	Baseline	Zero-shot	LLM performed the same or better than expert answers in 94% of cases for correctness, 77% for completeness, and 91% for conciseness
Janopaul-Naylor 2024^70^	Pan-cancer	Health Information	ChatGPT-4.0	Baseline	Zero-shot	Average scores: 3.9 (ChatGPT), 3.2 (Bing); DISCERN scores: 4.1 (ChatGPT), 4.4 (Bing)
Musheyev 2024^71^	Genitourinary	Health Information	ChatGPT-3.5; Chat Sonic; Microsoft Bing AI	Baseline	Zero-shot	AI chatbot responses had moderate to high information quality; Understandability was moderate; Actionability was moderate to poor; Readibility was low
Hermann 2023^72^	Gynecologic	Health Information	ChatGPT 3.5	Baseline	Zero-shot	Score of 1 for 34 questions (best); Score of 2 for 19 questions; Score of 3 for 10 questions; Score of 4 for 1 question
Davis 2023^73^	Genitourinary	Health Information	ChatGPT-3.5	Baseline	Zero-shot	14/18 were appropriate; Mean SD Flesch Reading Ease score: 35.5 (SD = 10.2)
Szczesniewski 2023^74^	Genitourinary	Health Information	ChatGPT-4.0	Baseline	Zero-shot	Treatment Modalities: 3/5; Average for Pathologies: 4/5; BPH: 3/5
Walker 2023^75^	Pan-cancer	Health Information	ChatGPT-4.0	Baseline	Zero-shot	Median EQIP score: 16 (IQR 14.5-18)/36; Agreement between guideline and LLM responses: 60% (15/25)
Patel 2023^76^	Pan-cancer	Health Information	ChatGPT-3.5, Curie, Babbage, Ada, Davinci	Baseline	Zero-shot	ChatGPT: 96%; Davinci:72%; Curie:32%; Babbage: 6%; Ada; 2%
Rahsepar 2023^77^	Lung	Health Information	ChatGPT-3.5; Google Bard	Baseline	Zero-shot	ChatGPT: 70.8% correct, 11.7% partiallly correct, 17.5% incorrect; Bard: 19.2% no answer, 51.7% correct, 9.2% partially correct, 20% incorrect
Kassab 2023^78^	Pan-cancer	Health Information	ChatGPT-3.5	Baseline	Zero-shot	Responses were accurate 86% of the time; 14% were innacurate; 0% harmful
Gortz 2023	Genitourinary	Health Information	SAP Conversational AI	Fine tuned	Zero-shot	78% of users did not need assistance during usage; 89% experienced an increase in knowledge about PC; 100% of users would like to reuse a medical chatbot in clinical settings
Koroglu 2023	Thyroid	Health Information	ChatGPT-3.5	Baseline	Zero-shot	Questions: Rater 1: 6.47+/-0.50; Rater 2: 6.18+/-0.96; Cases: Rater 1: largely correct, safe and usable; Rater 2: partially or moslty correct, safe and usable
Huang 2023^79^	Pan-cancer	Health Information	ChatGPT-4.0	Baseline	Prompt Engineered	Test: GPT 3.5 and 4 had scores of 62.05% and 78.77% respectively; Cases: GPT4 is able to suggest a personalized treatment to each case with high correctness and comprehensiveness
Holmes 2023^80^	Pan-cancer	Health Information	ChatGPT-3.5, ChatGPT-4.0, Google Bard, BLOOMZ	Baseline	Prompt Engineered	ChatGPT-4.0 outperformed all other LLMs and medical physicists on average; Accuracy improved when prompted to explain before answering
Yeo 2023^81^	Liver	Health Information	ChatGPT-3.5	Baseline	Zero-shot	Cirrhosis question accuracy: 79.1%; HCC question accuracy: 74%

**Figure 2.**
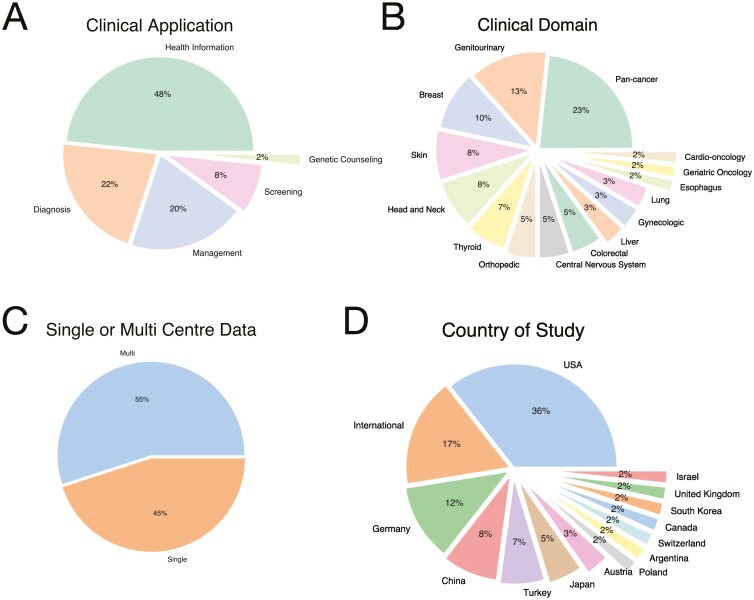
Proportion of all studies by A. clinical application, B. clinical domain, C. single or multi-center data, and D. country of study.

We observed that the majority of studies evaluated LLMs performance on oncology-related examinations as a health information resource(48%), followed by clinical diagnosis (22%) and management (20%) applications (Figure 2A). Across clinical specialties, LLMs were tested in diverse clinical oncology sites, most commonly in pan-cancer (23%), breast genitourinary (13%), breast (10%), skin(8%), and head and neck (8%) settings. The majority of studies (55%) evaluated LLMs based on test datasets sourced from multiple institutional sites compared to a single institutional site. These studies were primarily conducted by author teams from the United States (37%), followed by international settings (17%), Germany (12%), China (8%), and Turkey (7%).

**Figure 3.**
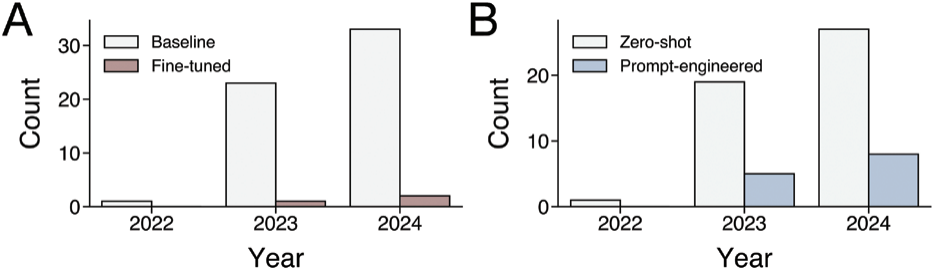
Number of studies that evaluated A. fine-tuning and B. prompt engineering methodologies to optimize chatbot medical accuracy.

The number of studies that evaluated the medical accuracy of LLMs in clinical oncology contexts significantly increased over time from 2022 to 2024 (Figure 3). Notably, the number of studies that fine-tuned LLMs (Figure 3A) and prompt-engineered LLMs (Figure 3B) also increased over time, starting from 2023 to 2024.

To highlight the benefits of LLM applications in clinical oncology, we observed that studies most commonly reported positive advantages that included their utility as a health information resource, their purported medical accuracy, the ability to reduce clinician workload, as well as improved accessibility and readability of generated information. However, these studies also noted several common disadvantages of these LLM applications, including the need to improve LLM-generated output reliability (consistency) as well as scope limitations that require clinician oversight.

## Discussion

Our scoping review of 60 studies highlights the role of LLMs in clinical oncology, particularly in applications of health information access, clinical diagnosis, and management. The utilization of ChatGPT-based models has been increasingly recognized for their potential to enhance medical accuracy and reduce clinician workload. These results underscore the transformative potential of LLMs in oncology, along with the necessity for ongoing enhancements to address their limitations.

We found that the literature on the utilization of LLMs as health information resources predominates their current application in clinical oncology, reflecting a pivotal shift toward integrating AI in patient education and engagement strategies. Notably, the proficient competency of LLMs to score well in oncology-related examinations, including questions sourced from professional society and established board examinations included in this review,^81,85^ underscores the utility of LLMs to serve as an information resource that can be consulted for clinical recommendations to in part inform clinical decision-making. For example, GPT-4 was able to achieve 79% accuracy on radiation-oncology in-training exams, and 86% on questions related to head and neck cancer. The proficiency of LLMs in delivering accurate, accessible, and readable information represents a significant advancement in medical informatics, where beyond clinical decision-making, can also help inform patient education and decision-making processes with clinician oversight.^46,59,81^ However, we caution against the accuracy of these chatbots as a patient-facing resource until further evaluation of medical and social biases is conducted.

This is especially crucial in oncology, where patients must comprehend complex treatment modalities and manage extensive, often daunting, care regimens.^84^ The effective use of LLMs in these contexts supports informed patient choices, enhances patient engagement, and improves compliance with treatment protocols. The reliability of the information provided by LLMs remains a commonly reported concern across studies included in this review.^85^ Among the 60 included studies, 25 (41.7%) reported biases and or hallucinations as a limitation. Inconsistencies and inaccuracies in the outputs—sometimes leading to information hallucinations—underscore the necessity for ongoing improvements and rigorous validation of these models to ensure accuracy and trustworthiness,^86^ as these directly impact patient care decisions and outcomes.

In addition to their role in health information dissemination, LLMs also hold significant potential in clinical diagnosis, as evidenced by 20% of the evaluated applications. LLMs can process vast amounts of medical data, including patient histories, imaging results, and laboratory findings, to assist clinicians in making accurate and timely diagnoses.^31,54,81,87^ This capability is particularly valuable in oncology, where early and precise diagnosis is crucial for effective treatment.^88^ By leveraging advanced algorithms and extensive medical knowledge bases, LLMs can identify patterns and correlations that may not be immediately apparent to human clinicians, contributing to potentially more precise diagnostic outcomes, reducing the likelihood of misdiagnoses, and enabling tailored treatment plans that improve patient prognosis.^13,39^ For instance, our review found that chatbots applied to diagnostic assessments from imaging reports in orthopedic,^28^ breast,^29^ and head and neck^31^ cancer contexts showed moderate to high agreement with clinician experts. Likewise, studies benchmarking LLM management recommendations applied to complex cases showed high concordance compared to multidisciplinary tumor board recommendations by clinicians in colorectal^44^ and breast^46^ contexts. Nonetheless, the integration of LLMs into diagnostic workflows must be approached with caution, ensuring that AI-driven recommendations are validated by clinical expertise and remain under regulatory oversight to maintain the highest standards of patient care.^13,89^

Furthermore, we found LLMs have shown significant potential in augmenting clinical management. These models can support clinicians in developing and monitoring treatment plans, managing medication regimens, and coordinating multidisciplinary care.^41,43,46,90^ In oncology, where treatment often involves complex, multi-faceted approaches, LLMs can enhance clinical decision-making by providing evidence-based recommendations and continuously updating care plans based on the latest medical research and patient data.^45,47,48^ Additionally, LLMs can improve patient management by facilitating communication and information sharing among healthcare teams, ensuring alignment with the patient’s treatment goals.^48,91^ This collaborative approach can lead to more cohesive and efficient care delivery, ultimately improving patient outcomes and satisfaction. As with their use in diagnosis, the application of LLMs in clinical management requires rigorous validation and oversight to ensure their reliability and effectiveness in real-world settings.

The application of LLMs in diverse clinical oncology settings reflects their broad potential and adaptability across different cancer care contexts. Our review found that LLMs were most commonly tested in pan-cancer settings, as observed in 23% of the included studies. This suggests that these models are seen as versatile tools capable of addressing general oncological needs across multiple cancer types. This wide applicability could be pivotal for developing generalized AI systems that can support decision-making and patient management on a broad scale. Additionally, the use of LLMs in common cancers including breast, skin, thyroid, urology, and head and neck cancers highlights their potential to address the unique challenges and treatment modalities specific to these types of cancer. Each of these areas has distinct diagnostic and therapeutic pathways, suggesting that LLMs may be effectively tailored to meet specific clinical requirements and improve care outcomes in speciality fields.^92^ The relatively lower application rates in prostate, colorectal, and bone cancers indicate potential areas for further research and development. Expanding the use of LLMs into these and other less common cancer types and facets of cancer care could ensure that the benefits of AI are more evenly distributed across all fields of oncology.^93,94^ To address these disadvantages, studies recommended the need for additional testing, continuing to evaluate performance in hold-out validation contexts, as well as improvements to medical accuracy and utility in diverse clinical scenarios.

Despite the challenges outlined, the benefits reported across studies suggest that LLMs can significantly enhance various aspects of oncology care, including diagnostics, patient management, and follow-up care. The ability of LLMs to process and analyze vast amounts of data can lead to more precise diagnostics and personalized treatment plans, potentially improving patient outcomes.^48^ However, the integration of these technologies must be approached with caution. The importance of clinician oversight cannot be overstated, as human expertise is crucial in interpreting AI suggestions and making final clinical decisions. This critical element was underscored in 8 (13.3%) studies, 4 of which explicitly mentioned the necessity for clinicians to finalize decisions.^38,39,51,52^

LLMs continue to undergo rapid development of capabilities and features that improve their ability to generate language suited for its intended purpose. The use of ambient AI scribes, which automate note generation around clinical encounters in real time, continues to grow in popularity.^95^ The use of prompt engineering has also shown particular promise; prompts that provide additional clinical context have been shown to generate treatment plans in concordance with cancer care guidelines,^47^ and chatbots have performed well in response to exam-style multiple-choice prompts.^96^ To this end, numerous publications have focused on benchmarking prompt engineering techniques,^97^ and educating healthcare professionals on best practices.^98,99^

Given the rapid developments of new versions of LLM chatbots trained on larger datasets with greater numbers of parameters, the hypothesis is that new versions of the same LLM would perform with higher medical accuracy compared to their predecessors in comparable evaluation scenarios. Indeed, in the subset of diagnostic^29,32^ and management^6,41,49,53^ scenarios that evaluated both GPT-4 and GPT-3.5, we observed that GPT-4 consistently demonstrated improved accuracy performance compared to its predecessor. Likewise, in the subset of health information evaluation scenarios that assessed both GPT-4 and GPT-3.5, we observed that GPT-4 usually demonstrated improved accuracy performance compared to its predecessor.^7,64,66,80^ Notably, Ostrowska et al observed that GPT-3.5 generated higher quality and more safety-aligned responses to medical information queries related to laryngeal cancer compared to GPT-4.^56^ We hypothesize that this finding is an exceptional observation that may not reflect the positive trend of improved medical knowledge and performance of GPT-4 compared to GPT-3.5. Further research is needed to systematically evaluate testing reliability to confirm if different models have unique deficiencies across different domains of medical knowledge.

Our scoping review has several limitations. Given the mixed performance metrics and benchmark datasets used across pilot evaluations of LLM medical accuracy, we were unable to define pooled measures across studies necessary to meta-analyze LLM performance. We underscore the importance of standardized data reporting in LLM and AI research through the use of established reporting guidelines, such as SPIRIT-AI for AI clinical trial protocols^100^ and CONSORT-AI for AI clinical trials.^101^ The studies included predominantly involved data from high-resource settings, primarily in the United States, which may limit the generalizability of the findings to lower-resource settings or diverse global populations. The reliance on published literature also raises concerns regarding publication bias, as studies with positive outcomes are more likely to be published. Furthermore, as with any rapidly evolving technology, the pace at which new research and LLM applications are developed may mean that some of the included studies do not reflect the most current state of the art. We note the over-representation of GPT-3.5 LLM among the included studies in this review and the potential for more recent chatbots, such as GPT-4, to perform more accurately due to its larger training dataset that comprises its knowledge base. Future research should focus on developing more robust LLMs capable of handling the variability and complexity of clinical data while maintaining the fidelity of the content generated.^49,75,77^ Furthermore, it is imperative to explore the ethical implications of using AI in patient interactions. Issues such as data privacy, the potential for bias in AI-driven decisions, and the impact of these technologies on the patient-clinician relationship must be critically assessed.^49,77^ Ensuring ethical standards and maintaining transparency in AI applications will be key to fostering trust and integrating these technologies into healthcare settings responsibly.

## Conclusion

LLMs in oncology represent a promising frontier in medical technology, with the potential to significantly enhance patient care and clinical efficiency. However, realizing this potential fully requires addressing current limitations through rigorous testing, validation, and ethical consideration. As LLMs continue to evolve, their integration into clinical practice must be approached with a strong emphasis on enhancing medical accuracy and ensuring patient safety, guided by empirical evidence and clinical expertise.

## Supplementary Material

oyaf038_suppl_Supplementary_Tables_2

oyaf038_suppl_Supplementary_Tables_3

oyaf038_suppl_Supplementary_Tables_1

## Data Availability

All data generated from this study are included in the study tables, figures, and supplementary tables. Inquiries can be directed to the corresponding author upon reasonable request.
